# Intestinal microbiota and melatonin in the treatment of secondary injury and complications after spinal cord injury

**DOI:** 10.3389/fnins.2022.981772

**Published:** 2022-11-09

**Authors:** Yiwen Zhang, Rui Lang, Shunyu Guo, Xiaoqin Luo, Huiting Li, Cencen Liu, Wei Dong, Changshun Bao, Yang Yu

**Affiliations:** ^1^Department of Human Anatomy and Histoembryology, School of Basic Medical Sciences, Southwest Medical University, Luzhou, China; ^2^Department of Neurosurgery, The Affiliated Hospital of Southwest Medical University, Luzhou, China; ^3^Key Laboratory of Medical Electrophysiology, Ministry of Education and Medical Electrophysiological Key Laboratory of Sichuan Province, Collaborative Innovation Center for Prevention of Cardiovascular Diseases, Institute of Cardiovascular Research, Southwest Medical University, Luzhou, China; ^4^Department of Pathology, People’s Hospital of Zhongjiang County, Deyang, China; ^5^Sichuan Clinical Research Center for Neurosurgery, The Affiliated Hospital of Southwest Medical University, Luzhou, China; ^6^Academician (Expert) Workstation of Sichuan Province, The Affiliated Hospital of Southwest Medical University, Luzhou, China; ^7^Neurological Diseases and Brain Function Laboratory, The Affiliated Hospital of Southwest Medical University, Luzhou, China

**Keywords:** spinal cord injury, melatonin, intestinal microbiota, secondary injury, complications, treatment spinal cord injury, treatment

## Abstract

Spinal cord injury (SCI) is a central nervous system (CNS) disease that can cause sensory and motor impairment below the level of injury. Currently, the treatment scheme for SCI mainly focuses on secondary injury and complications. Recent studies have shown that SCI leads to an imbalance of intestinal microbiota and the imbalance is also associated with complications after SCI, possibly through the microbial-brain-gut axis. Melatonin is secreted in many parts of the body including pineal gland and gut, effectively protecting the spinal cord from secondary damage. The secretion of melatonin is affected by circadian rhythms, known as the dark light cycle, and SCI would also cause dysregulation of melatonin secretion. In addition, melatonin is closely related to the intestinal microbiota, which protects the barrier function of the gut through its antioxidant and anti-inflammatory effects, and increases the abundance of intestinal microbiota by influencing the metabolism of the intestinal microbiota. Furthermore, the intestinal microbiota can influence melatonin formation by regulating tryptophan and serotonin metabolism. This paper summarizes and reviews the knowledge on the relationship among intestinal microbiota, melatonin, and SCI in recent years, to provide new theories and ideas for clinical research related to SCI treatment.

## Introduction

**FIGURE 1:**
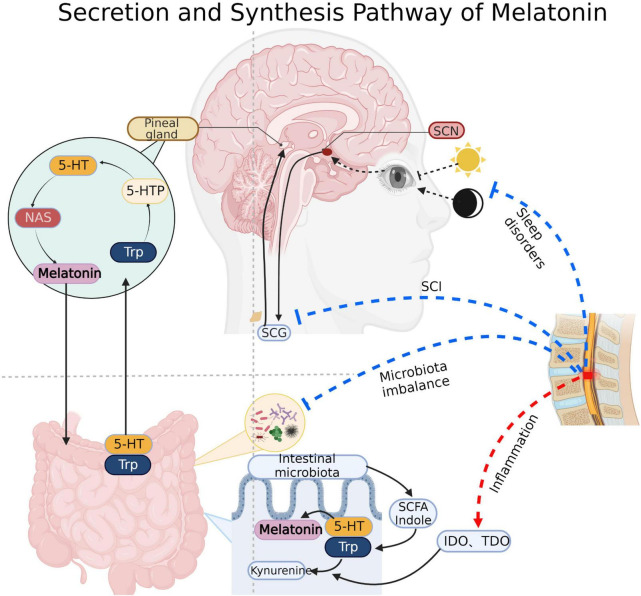
Melatonin synthesis: Pineal cells absorb tryptophan (Trp) and produce melatonin through intermediates such as 5-hydroxytryptophan (5-HTP), serotonin and N-acetyl-5-hydroxytryptamine (NAS). Substances such as indole and short chain fatty acids produced by gut microbiota also promote the synthesis of gut-derived melatonin. Light signal regulation of melatonin: Light signal received by the optic apparatus transmits to the supraoptic nucleus (SCN) of the hypothalamus, then it continues to transmit to the sympathetic cervical ganglion (SCG) through the brainstem and spinal cord, and finally arrived at the pineal gland to regulate the secretion of melatonin. Spinal cord direct damage, sleep disorders, microbiota imbalance and inflammatory will inhibit the synthesis and secretion of melatonin after SCI. IDO, Indoleamine 2, 3-dioxygenase; TDO, Tryptophan dioxygenase. Created with BioRender.com.

SCI is a highly disabling disease caused by various events, including traffic accidents, fall injuries, sports injuries, and tumors triggering spinal cord compression, traction, and contusion (Zhang et al., 2017). Besides the primary injury, the subsequent secondary injury caused by inflammation, edema, free radical peroxidation, local circulatory disorder, abnormal energy metabolism, electrolyte disorder, programmed apoptosis, nitric oxide and endogenous opioid peptides, and accumulation of excitatory neurotransmitters further exacerbate the damage to the spinal cord tissue (Anjum et al., 2020). Furthermore, multiple complications after SCI including respiratory and urinary tract infections, pressure ulcer, deep vein thrombosis and pulmonary embolism, neurogenic bladder, gastrointestinal dysfunction, sexual dysfunction and so on, have become significant causes of death and low quality of life in the recovery period (Rogers and Todd, 2016). The prognosis of SCI is usually related to the degree of primary injury and the treatment of segments, injury time, secondary injury and complications. Surgery to relieve compression after injury (within 24–36 h) is considered an effective and critically effective treatment to improve the prognosis of SCI (Badhiwala et al., 2021). In addition, hyperbaric oxygen, pulse electrical stimulation, mild hypothermia therapy, acupuncture, laser puncture, rehabilitation training, and other therapies could be used to assist in the recovery of function (Huang et al., 2020). Pharmacological treatment, neurotrophic factor therapy, cell transplantation therapy, gene therapy, and intervention in signaling pathways are thought to be potentially efficient in improving the prognosis of SCI, but more clinical trials are still needed to prove their effectiveness (Badhiwala et al., 2021). SCI has different pathological changes in various periods. Acute (= 2–48 h), and sub-acute (=14 days) after SCI, pathological changes such as vascular injury, ionic imbalance, free radical production, lipid peroxidation, accumulation of excitatory neurotransmitters, edema, necrosis and apoptosis occur at this stage (Faden et al., 1989; Oyinbo, 2011; Borgens and Liu-Snyder, 2012; Anjum et al., 2020). In the chronic SCI stage (> 1 month post-injury), characterized by chronic inflammation, axonal loss and maturation of glial scarring (Tran et al., 2018; Quadri et al., 2020). In addition, there are differences between acute and chronic inflammation. Following the primary injury, microglia at the injury site release proinflammatory and chemotactic factors, as well as recruit central granulocytes to mediate acute inflammation. At this time, M1 and M2 macrophages coexist (Zhou et al., 2014; Gensel and Zhang, 2015; Hellenbrand et al., 2021). In the chronic stage, M1 macrophages exist for a long time and mediate chronic inflammation with mature lymphocytes (Bastien and Lacroix, 2014; Schwab et al., 2014). Melatonin (N-acetyl-5-methoxy tryptamine) is an indole hormone secreted by the pineal gland. The pineal gland takes tryptophan in the blood as raw material and synthesizes 5-methoxy-n-acetamide through various enzymatic reactions (Vasey et al., 2021; Figure 1). Its secretion is regulated by circadian rhythm, inhibited during the day and active at night. In addition to the pineal gland, melatonin can also come from the intestines, skin, retina, bone marrow, platelets and other structures. However, it acts primarily locally and rarely throughout the whole body *via* blood circulation (Huether, 1993; Bubenik, 2002; Ren et al., 2017).

In terms of dosage, there is little difference between the physiological and pharmacological effects of melatonin, physiological dose provides plasma melatonin levels of the same order of magnitude as a nocturnal peak (less or around 100 pg/ml or 400 pM). Oral administration of 0.3 mg can reach the endogenous level at night (Dubocovich et al., 2010; Claustrat and Leston, 2015). In addition, continuous desensitization of receptors caused by excessive use of melatonin may affect sleep and circadian rhythm (Gerdin et al., 2004; Wurtman, 2006).

Melatonin which the CNS synthesizes has good blood-brain barrier permeability. It determines that melatonin can ensure sufficient concentration when interacting with other CNS parts through blood circulation. Melatonin is known to have a variety of biological functions, including circadian rhythm regulation, anti-inflammatory, free radical scavenging, edema reduction, suppression of the hypothalamic-pituitary endocrine axis, suppression of cancer, stimulation of mitochondrial biogenesis, immunomodulation, blood pressure regulation and the epigenetic regulation, and has therapeutic effects on a variety of secondary injuries after SCI (Giannoulia-Karantana et al., 2006; de Faria Poloni et al., 2011; Mazzoccoli et al., 2011; Xu L.X. et al., 2017; Chang et al., 2018; Onaolapo et al., 2020; Cho et al., 2021; Vasey et al., 2021). Melatonin can also regulate the intestinal biological clock and the types of intestinal microbiota, improve the metabolic disorder caused by intestinal microbiota imbalance after SCI, and indirectly play a role in the recovery of function and the treatment of complications (Paulose et al., 2016). The route of administration and detection method are simple and convenient, which are conducive to investigation and evaluation. The exogenous melatonin can be administered orally or intravenously and its concentration can be assessed by blood, saliva and so on. It is considered a more convenient and accessible approach to provide greater comfort to patients and improve their quality of life (Dermanowski et al., 2022).

The intestinal microbiota can act on the CNS through the microbial gut-brain axis. The normal microbial ecology of the intestinal tract helps the human body generate a range of vitamins, engage in carbohydrate, protein, and lipid metabolism, promote trace element absorption, proper intestinal peristalsis, feces excretion, and prevent the growth of dangerous bacteria (Blaut, 2013). The brain-gut axis seems to provide new discoveries in some CNS diseases. The brain can transmit information from top to bottom *via* the autonomic nervous system, the gut nervous system, the hypothalamus pituitary adrenal axis (HPA), and the meningeal lymphatic vessels (Yuan et al., 2021). many neurological diseases, such as depression, multiple sclerosis (MS), Alzheimer’s disease (AD), Parkinson’s disease, and others, can cause changes in the structure of the intestinal microbiota (Joscelyn and Kasper, 2014; Williams and Murray, 2015; Sun et al., 2018; Anderson et al., 2019). In addition, the intestine can also transmit signals from bottom to top through the vagus and immune pathways (Yuan et al., 2021). Patients with Parkinson’s disease have higher levels of lipopolysaccharide (LPS) and lower levels of short-chain fatty acid (SCFA) in the intestine, leading to chronic inflammation in the CNS and promoting disease progression (Keshavarzian et al., 2015; Perez-Pardo et al., 2019). Increasing the percentage of intestinal regulatory T (Treg) cells but decreasing interleukin-17-gamma-delta T cells can alleviate nerve defects and anti-inflammation after stroke (Lee et al., 2020). Intestinal microbiota promotes the occurrence of the intracranial aneurysm by regulating plasma taurine and fatty acid levels (Li et al., 2020). It also improves neuronal degeneration, brain edema and blood-brain-barrier damage by increasing the level of glucagon-like peptide 1 (Li et al., 2018), and improves spatial learning through SCFA after traumatic brain injury (Opeyemi et al., 2021). In addition, the secretion of intestinal bacterial amyloid protein and lipopolysaccharide (LPS) promotes neuroinflammation and causes neuronal death in AD (Pistollato et al., 2016; Kesika et al., 2021). Some studies suggest that regulating stress state and HPA through fecal bacteria transplantation may be significant in treating depression (Liang et al., 2018).

SCI is usually accompanied by the destruction of intestinal ecology through mechanisms associated with disruption of signals from higher control center, autonomic nerve dysfunction, intestinal barrier permeability change, mesenteric lymph node immune regulation, and long-term use of antibiotics (Cervi et al., 2014; Kigerl et al., 2016; Tate et al., 2016; Jing et al., 2019). Studies have shown that intestinal microbiota modulates the functional recovery after SCI through SCFA and the immune system (Jing et al., 2021b; Rodenhouse et al., 2022). Alternatively, there is evidence that fecal flora transplantation (FMT) may be effective in improving complications such as gastrointestinal dysfunction, bloating and constipation after SCI (Jing et al., 2021a). Furthermore, intestinal microbiota could directly or indirectly participate in the synthesis of melatonin. Melatonin is derived from serotonin (5-hydroxytryptamine, 5-HT). Intestinal bacteria may directly produce SCFAs, which stimulate the production of 5-HT. Then, the 5-HT is successively converted into N-acetyl-5-hydroxytryptamine (NAS) and melatonin by arylalkylamine N-acetyltransferase (AANAT, EC: 2.3.1.87) and acetylserotonin O-methyltransferase (ASMT, EC: 2.1.1.4) (Benabou et al., 2017; Figure 1).

As a result, a bidirectional link is observed, in which changes in intestinal microbiota influence the regulation of the melatonergic system, and treatment of melatonin cause changes in microbiota, with both factors contributing to the pathobiology and treatment of SCI. This review aims to describe the potential links among gut microbiota, melatonin, and SCI and to explore the synergistic effects of melatonin and the regulation of gut microbiota in improving the prognosis of SCI.

## The relationship between melatonin and intestinal microbiota

### Melatonin regulates the recovery of intestinal microbiota disorders

#### Melatonin affects the composition of intestinal microbiota

Melatonin regulate the intestinal microbiota, which improve the reduction of intestinal operational taxonomic unit (OTU), increase ACE index and Shannon index, decrease Simpson index, increase the diversity and abundance of intestinal microbiota, increase the abundance of firmicum and decrease the abundance of Bacteroides (Ren et al., 2018; Gao T. et al., 2020; Kim et al., 2020). Melatonin also increases Akkermansia flora’s abundance (Hagi and Belzer, 2021). In the SCI mouse model, melatonin reduce the abundance of Clostridium and increase the abundance of Lactobacillus (Jing et al., 2019). Also, melatonin could increase the abundance of Myxobacteria and Streptococcus mucophaphagus (Park et al., 2020), protect intestinal barrier and reduce intestinal inflammation. In addition, melatonin enhances the abundance of bacteria and decrease the ratio of firmicum to Bacteroides in a high-fat diet-induced obesity mouse model (Xu P. et al., 2017; Yin et al., 2018). In the ochratoxina induced liver injury model, melatonin increase the abundance of Bacteroides, verrucous microorganisms and actinomycetes, while decrease the abundance of firmicum and Lactobacillus (Zhang H. et al., 2021; Zhao et al., 2021). In terms of microbial metabolism, melatonin significantly reduces the relative abundance of the microbiome in the cysteine and methionine metabolism and peptidoglycan biosynthesis pathway in the restraint stress mouse model, while increasing the relative abundance of the microbiome in the tryptophan metabolism, aminobenzoic acid degradation, renin angiotensin system, γ-aminobutyric acid ergic synapses and type II diabetes pathway (Lin et al., 2021).

#### Protective effect of melatonin on intestinal barrier

There are three interconnected barriers in the intestine, a biological barrier made up of the intestinal microbiota; an intestinal immune barrier made up of the intestinal lymphatic system; and a physical barrier comprising the voluntary movement of the intestine and the mucosal epithelial system (Gao et al., 2019). The fluorescein isothiocyanate labeled dextran experiment proved that the intestinal permeability in stress state would increase (Jing et al., 2019). Under stress, the number of intestinal goblet cells and intestinal gland proliferating cell nuclear antigen positive cells decreased, which affect mucus secreted and intestinal cells proliferated, and then the repair effect of intestine barrier was damaged. Meanwhile, the activation of nuclear factor-kappaB (NF-κB) pathway induced autophagy and eventually damages the intestinal mechanical barrier (Gao et al., 2019).

The expression of tight junction-related proteins claudin-1, occludin and zonula occludens-1 (ZO-1) decreased in stress models such as SCI and sleep deprivation, and melatonin is effective in improving the reduction of occludin and ZO-1 (Gao et al., 2019; Jing et al., 2019). It suggested that melatonin may strengthen the tight connection between cells, regulate intestinal permeability, repair and maintain the stability of intestinal mechanical barrier, which effectively prevent toxins and other bacterial metabolites in the intestine from entering the blood and cause cascading chain inflammatory reaction. A similar effect was also observed in the oxazolone induced colitis model (Zhao et al., 2021), indicating that melatonin’s preservation of the intestinal barrier was not confined to stress damage and that its barrier protective role had a broader application. In addition, melatonin accelerates the emptying effect of the gastrointestinal tract in mice after SCI (Jing et al., 2019), demonstrating that melatonin enhances gastrointestinal function, promotes peristalsis and strengthens the mechanical barrier.

#### Melatonin regulates intestinal immune function and down-regulates inflammatory response

In prolonged paradoxical sleep deprivation mouse model, with the activation of the HPA, a large amount of cortisol were released into the systemic circulation, which would mediate the disturbance of intestinal microbiota (Galvão Mde et al., 2009), stimulate a cascade of transcription factors and associated signaling pathways. Cortisol activated the protein expression of the signal transducer and activator of transcription 3 (STAT-3)/activator protein 1 (AP-1)/NF-κB pathway, such as phosphorylated-signal transducer and activator of transcription-3 (p-STAT3), AP-1, p-P65 and phosphorylated-inhibitor of nuclear factor-kappaB (p-IκB), while melatonin decreased the expression of the above proteins, suggesting that melatonin suppressed inflammation and autophagy by inhibiting NF-κB pathway (Gao et al., 2021). Research has shown that the stress induces changes in intestinal permeability leading to the secretion of LPS by the intestinal microbiota which in turn activates the TLR4 inflammatory signaling pathway to release of several proinflammatory cytokine such as interleukin-1β (IL-1β), IL-6 and tumor necrosis factor-α (TNF-α) (Ghareghani et al., 2018). In a mouse model of colitis, melatonin inhibited the increase of IL-1β, IL-17 and TNF-α, whereas in TLR4 knockout mice melatonin has been found to fail to inhibit effectively the increased levels of IL-1β, IL-17, and TNF-α (Kim et al., 2020), suggesting that melatonin may suppress the LPS/TLR4 signaling pathway by inhibiting expression of pro-inflammation cytokines. In addition, the expression of anti-inflammatory factors (IL-5, IL-10, IL-22) was regulated by administration of melatonin on in a stress model (Gao et al., 2019, Gao K. et al., 2020; Lin et al., 2021), and further inhibit intestinal inflammation. It was demonstrated that in a mouse model of SCI, melatonin displayed anti-inflammatory effects by reducing the upregulation of IL-17, interferon-gamma (IFN- γ) and monocyte chemoattractant protein-1 (MCP1) (Jing et al., 2019). In the oxazolone induced colitis model, melatonin could inhibit the production of IL-5, IL-13 and their mRNA in type 2 innate lymphocytes (ILC2s) and affect the type 2 immune response (Zhao et al., 2021). Ghareghani et al. (2018) and Xiong et al. (2022) demonstrated that melatonin modulates intestinal immune function through the expression of nod-like receptor-related genes and TLRs. By differential expression gene (DEG) and gene ontology (GO) analysis, *Tlr1*, *Tlr2*, *Tlr7*, *Cd14*, *Naip*, *Cxcl8* and *Nlrp1*, *Nlrp3* gene expression levels were found to be significantly increased in ASMT (a key enzyme in the acetyl serine synthesis melatonin pathway) overexpressing transgenic sheep (Li et al., 2021). It is suggested that melanin plays an important role by regulating anti-inflammatory related factors and signaling pathways. In general, melatonin can regulate the immune response by regulating intestinal TLRs and nod like receptors, and affect the type 2 immune response by inhibiting ILC2s. It also inhibits the STAT-3/AP-1/NF-κB pathway, down-regulating the release of inflammatory factors and up-regulating anti-inflammatory factors, suppressing the intestinal inflammatory response and setting the stage for ecological recovery of the microbiota.

### Intestinal microbiota affects melatonin secretion

Melatonin is abundant in the gastrointestinal tract, with approximately 10–100 times more melatonin in the gastrointestinal tissues than in the blood and 400 times more in the gut than in the pineal gland (Huether, 1993; Bubenik, 2002). Intestinal microbiota can affect melatonin secretion through a variety of mechanisms. High dietary fiber diet promoted the increase of intestinal short chain fatty acid concentration and 5-HT concentration in serum and intestinal mucosa, and promoted the up-regulation of tryptophan hydroxylase (TPH1) mRNA level (Zhuo et al., 2021). Besides, intestinal microbial metabolites, short chain fatty acids such as propionic acid and butyric acid, and tryptophan metabolite indoles can promote the production of 5-HT, which in turn further affects melatonin secretion *via* AANAT and ASMT (Gao et al., 2018; Figure 1).

Intestinal microbial sulfatase can promote melatonin secretion (Ervin et al., 2020). The experiments in zebrafish have proved that probiotics and dark conditions can increase the mRNA expression of melatonin receptor genes, mainly melatonin-1 related receptor genes (*Mtnr1ba* and *Mtnr1bb*) (Lutfi et al., 2021). The disturbance of intestinal microbiota affect the absorption of vitamins, which cause deficiencies in enzyme cofactors. In addition, the disruption of intestinal microbiota induces intestinal inflammatory reaction. Inflammatory and deficiencies of enzymatic cofactors would trigger the kynurenine metabolic pathway, promoting the synthesis of tryptophan into kynurenine and inhibiting the synthesis of tryptophan into 5-hydroxytryptamine and melatonin (Rudzki et al., 2021).

## Spinal cord injury and intestinal microbiota

The imbalance of intestinal microbiota has been proved to be related to various CNS diseases. Firmicutes and Bacteroides are two phylum of intestinal microbiota, both of them contain probiotics and opportunistic pathogens, which have dual effects on human body (Wexler and Goodman, 2017; Heintz-Buschart and Wilmes, 2018). Intestinal microbiota can produce short-chain fatty acids by metabolizing dietary fiber (Markowiak-Kopeć and liżewska, 2020). Previous studies found that obese people have higher level of Firmicutes and lower level of Bacteroides (Ley et al., 2005, 2006). In addition, another study found that obese people have higher levels of SCFA (Schwiertz et al., 2010), proving that the level of SCFA is directly proportional to Firmicutes.

Studies have confirmed that intestinal microbiota is disordered after SCI, which is manifested by the decrease of the abundance of Firmicutes, with or without the increase of bacteroides, resulting in the change of the ratio of Firmicutes to Bacteroides, this change in microbiota structure affects the production of short-chain fatty acids (Gungor et al., 2016; Zhang et al., 2018; Jing et al., 2019; Bazzocchi et al., 2021; Rodenhouse et al., 2022), this appears to be associated with severe inflammation and complex infection after SCI. There are significant differences in intestinal microbiota composition in patients after SCI in the acute and chronic stages. There is greater abundance of genus Sutterella in the acute stage, it may be related to more severe inflammation (Santoru et al., 2017). In the chronic stage, it had lower abundances of the Burkholderiaceae family. But with the increase of exercise, the abundance of Burkholderiaceae increases. It suggests that long-term inactivity after SCI may lead to this difference (Li et al., 2022). The changes in intestinal microbiota after SCI are related to the degree of injury. In American Spinal Injury Association Impairment Scale (AIS) A or B patients, the abundance of lactobacillus is higher, and in AIS C or D patients, Bacteroides, Faecalibacterium, Lachnospiraceae are more abundant (Bazzocchi et al., 2021). A study of patients with complete spinal cord injury (CTSCI) and incomplete spinal cord injury (ITSCI) has found that the abundance of Synergistetes is significantly different between CTSCI and ITSCI. Coriobacteriae, Eubacterium, and Cloacibacillus is more abundant in CTSCI, Lactobacillaceae, Lachnospiraceae, Eubacteria, Clostridium, and Sutterella is more abundant in ITSCI (Yu et al., 2021). Different levels of SCI will also lead to the different intestinal microbiota. Zhang et al. (2018) found that the abundance of Bacteroides is higher in quadriplegia patient, while Acidaminococcaceae, Blautia, Porphyromonadaceae, and Lachnoclostridium is higher in paraplegia patient. Another study pointed out that the Firmicutes in T10 is low, while actinomycetes in T4 and T10 are increased (Du et al., 2021).

### The causes of intestinal microbiota imbalance after spinal cord injury

#### Control interruption of advanced center, autonomic nerve function, and intestinal nerve function disorder

The regulation of intestinal function is actually co-regulated by sympathetic, parasympathetic and enteric nervous system (ENS) (Furness et al., 2014). After SCI, the injured ascending and descending neurons cannot dominate the lower neurons below the injured segment, nor can they transmit the information of lower neurons to the brain. The interruption of the control of the advanced center leads to the loss of gastrointestinal and bladder sympathetic control, smooth muscle dysfunction, impaired intestinal movement, fecal retention or incontinence (Holmes et al., 2020), and these variables are maintained throughout the acute to chronic phase of SCI. It has been studied to exclude the influence of sympathetic and parasympathetic on intestinal tract by injuring the high level thoracic spinal cord, in the mice with T8 SCI, it found that the number of nitroergic nerve cells decreased, and the number of acetylcholine decreased although the proportion of acetylcholinergic nerve did not change significantly (Lefèvre et al., 2020). Another study found that nitroergic and cholinergic neurons decreased in the mice with T3 SCI (White et al., 2020). These results suggest that the ENS is damaged after SCI, which lead to the destruction of colonic content secretion, propulsion and local reflex regulation related to colonic segmentation (Furness et al., 2014).

#### Immune regulation and inflammation of mesenteric lymph node gut-associated lymphoid tissue

Gut-associated lymphoid tissue (GALT) is innervated by the sympathetic nerve of the spinal cord. The loss of control of the sympathetic nerve destroys the immune homeostasis of GALT after SCI (Straub et al., 2006), further affecting the regulation of the intestinal immune system on the intestinal microbiota. Kigerl et al. (2016) found that the expression of TNF, IL-10, IL-1β, and transforming growth factor-β (TGF-β) is increased in mesenteric lymph nodes on the third day after SCI, and Li et al. (2022) found that different flora changes in the feces of patients in the acute stage of SCI compared with those in the chronic stage. In 8-week SCI animals, it has been found that IL-1β is significantly associated with 23 OTUs, and 12 OTUs were found to be significantly correlated to IL-12. Although macrophage inflammatory protein 2 is especially related to the difference in microbial diversity, no significant OTU is detected (O’Connor et al., 2018). It indicates that the occurrence of acute and chronic inflammation after SCI mediates the destruction of intestinal ecology.

#### Changes in intestinal epithelial barrier dysfunction

Intestinal epithelial barrier dysfunction has proved after SCI (Liu et al., 2020, 2021). The change leads to more opportunities for bacteria and their metabolites to enter the circulation, cause enterogenous infection, and further lead to the disorder of flora. Studies have shown that SCI affects the intestinal epithelial barrier and induces intestinal disturbances, leading to delayed motor recovery in mice (Kigerl et al., 2016).

#### Long-term use of antibiotics

Numerous studies have reported that many antibiotics exert anti-inflammatory and neuroprotective effects in different central and peripheral nervous system disorders, including SCI. But long-term use of antibiotics may further aggravate the disturbance of intestinal microbiota after SCI, inducing the increase of firmicum, Proteus and Actinobacillus the decrease of Bacteroides, and affecting the functional recovery after SCI (Jing et al., 2019; Bazzocchi et al., 2021; Rodenhouse et al., 2022). However, as an antibiotic, dimethylaminocycline has largely ignored its negative effects on intestinal microbiota and systemic immune response after SCI, which may be related to its direct anti-inflammatory, anti-oxidation, anti-anxiety, and neuroprotective properties (Schmidt et al., 2021).

### Improving the dysfunctional flora structure is beneficial to the functional recovery after spinal cord injury

#### Short-chain fatty acid regulate the inflammatory process and inhibit the neurotoxicity and secondary injury of microglia

**FIGURE 2:**
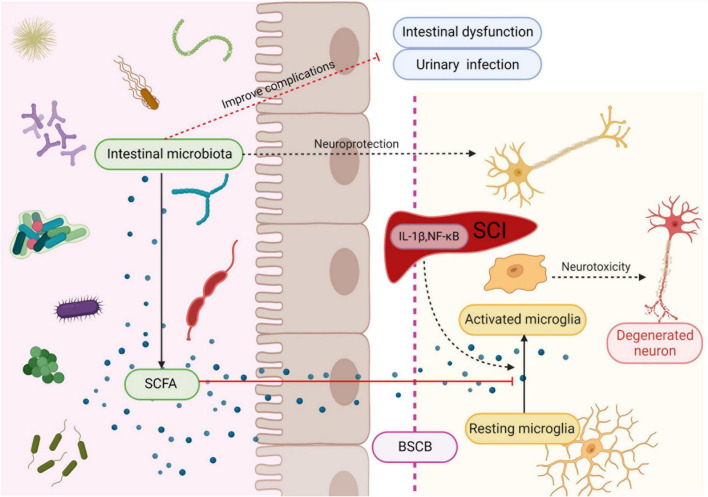
Intestinal microbiota protects neuron and improves complications after SCI. Microbiota secretes SCFA to regulate maturation and activation of microglia, thereby suppressing their neurotoxicity. Created with BioRender.com.

Microglia can rapidly transform into activated macrophages and produce toxic molecules that mediate lipid peroxidation, eventually leading to subsequent tissue damage and axonal contraction after SCI (Busch et al., 2009; Kigerl et al., 2009). Moreover, glial cells increase pain hypersensitivity by releasing various signal molecules, such as proinflammatory cytokines (DeLeo and Yezierski, 2001; Watkins et al., 2001; Hanisch, 2002). Short-chain fatty acids have strong anti-inflammatory effects on macrophages (Park et al., 2005; Chen et al., 2007) and inhibit CNS inflammation through the brain-gut axis. Patients with SCI have significantly lower levels of butyric acid producing bacteria, and the decreased butyric acid levels lead to reduce inhibition of microglia, resulting in microglia-mediated neurotoxicity, which may also be responsible for the persistent triggering of neuropathic pain. Low butyric acid levels may affect long-term functional recovery after SCI (Gungor et al., 2016). Another study demonstrated that probiotic-transplanted mice could reduce inflammatory response by increasing the production of SCFAs, which modulate gut immunological and barrier functions by inhibiting NF-κB signaling (Jing et al., 2021b; Figure 2). At the early stage of SCI, the level of Firmicutes decreases and Bacteroides increases, which affect the production of SCFA (Jing et al., 2021a). However, most studies of FMT for SCI have chosen in the chronic phase of SCI. Therefore, the alteration of intestinal flora after the intervention of intestinal flora in the acute phase of SCI need to be studied in depth.

#### Adjustment of flora structure to improve the function after spinal cord injury

It is suggested that probiotic transplantation to reconstitute the intestinal microbiota is beneficial in promoting functional recovery after SCI. In mice involved in the transplantation of probiotics to reconstitute the gut microbiota after SCI, the action evoked potential (MEP) amplitude was significantly higher in the probiotic-transplanted mice than in the SCI group. In addition, the number of NeuN + cells, NF-200 -positive cells, and the expression of synapsin increased in probiotic-transplanted mice, which suggested that probiotic transplantation may promote the survival of neurons and axonal regeneration after SCI (Jing et al., 2021a; Figure 2). On the other hand, transplantation of probiotics before SCI could reduce the loss of function after injury, and can effectively improve the flora structure disorder caused by antibiotics, which is considered to be positive significance for the recovery of neural function after SCI (Rodenhouse et al., 2022).

### Intestinal microbiota contributes to the treatment of autonomic nervous function complications

**FIGURE 3:**
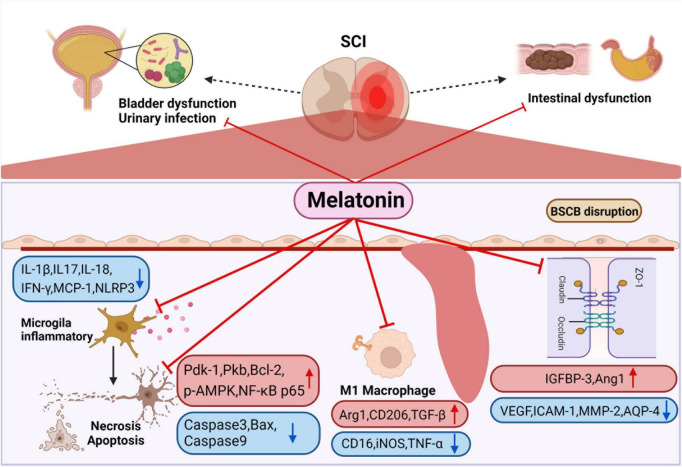
Smooth muscle dysfunction leads to constipation and fecal incontinence after SCI. Melatonin can enhance intestinal motility to promote emptying. It can also improve colonizing dominant bacteria abundance to reduce the probability of urinary infection. Melatonin inhibits the activation of M1 macrophage after SCI, the release of a variety of inflammatory factors are suppressed, and at the same time regulates apoptosis-related proteins to inhibit the apoptosis of nerve cells. In addition, melatonin regulates channel-related proteins to maintain the stability of blood-spinal cord barrier and reduce edema. Created with BioRender.com.

There are many related chronic complications after SCI, such as intestinal dysfunction, bladder dysfunction and sexual dysfunction (Figure 3), severely impair the quality of life. Studies have shown that compared with other complications, patients with SCI want to solve the problem of intestinal dysfunction (Glickman and Kamm, 1996; Lynch et al., 2001; Anderson, 2004; Zhang et al., 2018). Neurogenic motor gut dysfunction usually occurs after SCI, and it can be further classified into upper motor meta gut syndrome (UMN) and lower motor gut syndrome (LMN). In the mouse model, the mice that recovered the structure of intestinal microbiota after fecal microbiota transplantation showed faster gastrointestinal transit through barium gavage followed by X-ray imaging (Jing et al., 2021b).

After SCI, neurogenic bladder would also occur, resulting in urinary retention and incontinence, which further cause urinary system infection. It has been reported that the annual urinary tract infection rates of upper motor neuron intestinal syndrome group and lower motor neuron intestinal syndrome group were 3.0 ± 1.8 and 2.6 ± 1.8, respectively (Gungor et al., 2016). It has been proved that the transplantation of intestinal microbiota could improve refractory urinary tract infection (Tariq et al., 2017; Biehl et al., 2018; Magruder et al., 2019, 2020), which may be related to the change of dominant bacteria inhibiting the growth and colonization of pathogenic bacteria, but it still needs more in-depth research and prospective studies to explore the molecular mechanisms and confirm. C. J. Worby summarized that the influence of intestinal microbiota of urinary tract infection may come from three aspects. Firstly, the causative organisms of urinary tract infections come from the gut microbiota. Furthermore, a specific gut microbiota provides a suitable living environment for urinary tract infections. Eventually disturbances in the gut microbiota modulate the immune system (Worby et al., 2022). Therefore, improving the gut microbiota positively affects urinary tract infections.

## The relationship between spinal cord injury and melatonin

### Effect of spinal cord injury on melatonin secretion

SCI in different spinal cord segments lead to significantly disparate degrees of melatonin secretion. Studies have shown that the level of cortisol in thoracic SCI was higher, and the level of melatonin in cervical SCI was lower (Thøfner Hultén et al., 2018). It was found that melatonin levels were significantly lower in patients with completed cervical SCI than in the thoracolumbar SCI group and the normal group. Furthermore, melatonin levels appear to differ between patients with complete and incomplete SCI, but *in vivo* and *in vitro* experimental verification is lacking, and this hypothesis requires further experimental validation (Whelan et al., 2020). It has been previously confirmed that the secretion rhythm of melatonin is related to the suprachiasmatic nucleus (SCN) of the hypothalamus (Cardinali and Pévet, 1998). The nerve from SCN to the pineal gland passes through the upper part of the cervical spinal cord and connects with the preganglionic cells of the sympathetic cervical ganglion (SCG) (Møller and Baeres, 2002), which indicates that melatonin secretion is related to cervical spinal cord segments (Figure 1).

In addition, the change of melatonin secretion level may be related to sleep disorders after SCI (Hultén et al., 2020). An investigation on sleep disorders in patients with cervical myelopathy reported that spinal cord compression and area reduction were independent risk factors for sleep disorders. Therefore, patients with compression SCI caused by spinal canal stenosis are prone to sleep disorders, and subsequently affect melatonin secretion (Kim et al., 2021).

### Melatonin has a beneficial effect on the treatment of spinal cord injury

Melatonin has been proved by many studies to improve motor function recovery after SCI (Shen et al., 2017; Jing et al., 2019; Li et al., 2019; Wang K. et al., 2019; Xu et al., 2019; Gao K. et al., 2020; Majidpoor et al., 2020; Yang et al., 2020; Bi et al., 2021a), which is closely related to the anti-inflammatory, anti-apoptotic, tissue edema reducing, blood spinal cord barrier (BSCB) protection and nerve remodeling functions of melatonin (Figure 3). It was demonstrated that melatonin treatment significantly reduced the relative abundance of Clostridium perfringens, markedly increased the relative abundance of Lactobacillus, and dramatically improved function after SCI (Jing et al., 2019). In addition, melatonin can be used in combination with various treatment methods to treat SCI. For instance, polylactic acid glycolic acid copolymer sustained-release microspheres with melatonin and laponite hydrogel for mixed injection to treat SCI (Zhang M. et al., 2021). The use of melatonin in extracellular vesicles of stem cells reduces the levels of N6 methyladenosine modification (m6A) and methyltransferase 3 (METTL3) in SCI, and enhances the stability of USP29mRNA in MSCs, which also promote stem cell proliferation, differentiation, neurosphere formation and secretion of neurotrophic factors (Li et al., 2017; Zheng et al., 2017). In general, melatonin plays the role of anti-inflammatory, anti-apoptotic, tissue edema reducing, blood polar core barrier protection and nerve remodeling functions in acute and sub-acute stages.

#### Neuroprotective function of melatonin

Melatonin was effective in ameliorating neuronal apoptosis and consequently protecting neurons after SCI (Jing et al., 2017; Shen et al., 2017; Zheng et al., 2017; Li et al., 2019; Xu et al., 2019; Gao K. et al., 2020; Bi et al., 2021a). It has been found that melatonin treatment significantly increased the expression of apoptosis-related proteins, including phosphatidylinositol 3 kinase (PI3K) p85 and PI3K downstream proteins (phosphoinositide-dependent kinase-1, protein kinase B and NF-κB p65). The up-regulation of PI3K downstream protein was inhibited after using melatonin inhibitor luzindole, which indicates that melatonin inhibits apoptosis after SCI by activating PI3K pathway (Bi et al., 2021a,b). In addition, some studies found that melatonin down regulated the expression of Caspase3 and caspase9 after SCI, and the expression levels of autophagy marker protein (beclin-1), LC-3b and SITR1/AMPK signal pathway related proteins (SIRT1 and p-AMPK) increased significantly. The above effects disappeared after using SIRT1 inhibitor EX527, which indicates that melatonin can regulate autophagy and apoptosis through SIRT1/AMPK (Gao K. et al., 2020). Li et al. (2019) also demonstrated that melatonin enhances the autophagy of spinal cord neurons through PI3K/AKT/mTOR signaling pathway. Furthermore, melatonin significantly increased p-LRP6, β-catenin and LEF-1, the level of pro-apoptotic protein Bax was down-regulated, and the anti-apoptotic protein Bcl-2 was up-regulated, indicating that melatonin *via* Wnt/β- Catenin signaling pathway to regulate autophagy and apoptosis (Shen et al., 2017; Figure 3).

#### Melatonin down regulates the inflammatory response after spinal cord injury

Melatonin treatment can significantly reverse the expression of inflammatory related factors after SCI, such as IL-17 and IFN-γ, MCP-1 (Jing et al., 2019), NOD-like receptor protein 3 (NLRP3), IL-1 β, and caspase-1 (Xu et al., 2019; Yang et al., 2020). Some studies have pointed out that melatonin can inhibit inflammation after SCI *via* TLR4/NF-κB and NOX2/TXNIP signaling pathways (Paterniti et al., 2017; Majidpoor et al., 2020). Majidpoor et al. (2021) found that melatonin decreased the activities of NLRP3, apoptosis associated spotted protein and caspase-1, down regulated the level of IL-1 β and IL-18. The sensitivity of different segmental injuries to melatonin treatment is different. Among them, T6 is the most sensitive to melatonin treatment, except for T6, melatonin treatment does not affect the level of NLRP3 after SCI. IL-18 does not change only after melatonin treatment for T1 segmental injury (Majidpoor et al., 2021), which is undoubtedly interesting, but the reasons for this deserve further study.

However, few studies are concluding that melatonin has different sensitivity in different segments, which still needs to be further verified by other experiments. Zhang et al. (2019) found that melatonin treatment after SCI reduced M1 microglia markers (CD16, iNOS, TNF-a), but increased the levels of the M2 microglia markers (Arg1, CD206, TGF-β), which proves that melatonin promotes the differentiation of macrophages toward M2 type after SCI. In addition, melatonin inhibits the aggregation of microglia and thus the activation and proliferation of microglia and astrocytes (Paterniti et al., 2017; Yang et al., 2020; Figure 3).

#### Melatonin improves local blood circulation and edema, and maintained the stability of the blood spinal cord barrier

Studies have shown a reduction in the endothelial marker protein CD31 after SCI, suggesting damage to the blood-spinal cord barrier, and administration of melatonin increased the proportion of CD31-labeled vessels, suggesting that melatonin is effective in reducing the permeability of the blood-spinal cord barrier after SCI (Jing et al., 2017). Wang K. et al. (2019) proved that melatonin enhance the level of angiopoietin 1 (Ang1), reduce the expression of vascular endothelial growth factor (VEGF), adhesion factor intercellular adhesion molecule 1 and BSCB related factor matrix metallopeptidase 2 through insulin-like growth factor binding protein-3 (IGFBP-3), indicating that melatonin can maintain the stability of BSCB through interaction with IGFBP3 (Figure 3). Besides this, studies in animals have shown that melatonin administration reduces tissue edema after SCI. Xu et al. (2019) demonstrated that the water content of spinal cord tissue decreased significantly after melatonin administration and that melatonin downregulated the expression of the aquaporin aquaporin-4 (Li et al., 2014; Liu et al., 2015).

#### Melatonin prevents the down-regulation of neuroplasticity after spinal cord injury

Melatonin treatment after SCI significantly increased the mRNA and protein expression levels of growth associated protein-43 (GAP-43), synapsin1, Neurofilament 200 (NF200) and Postsynaptic density protein 95 (PSD-95) (Bi et al., 2021a), which play an important role in the process of neuronal remodeling. Moreover, brain-derived neurotrophic factor (BDNF) is the neuronal plasticity promoter, which affects neuronal remodeling through synapsin1 and GAP-43 protein. After SCI, the expression of BDNF, synapsin1 and GAP-43 in the spinal cord decreases, while melatonin can partially reverse the decrease of them, suggesting that melatonin has a protective effect on neural plasticity after SCI (Jing et al., 2017).

## Conclusion

The complications after SCI greatly impact quality of life and life expectancy, especially constipation and fecal incontinence caused by gastrointestinal and bladder dysfunction (Glickman and Kamm, 1996; Lynch et al., 2001; Anderson, 2004; Zhang et al., 2018). The intestinal microbiota is closely related to gastrointestinal dysfunction. After SCI, the loss of advanced central control of the sympathetic nerve, the destruction of intestinal immune homeostasis, the impairment of intestinal barrier function, inflammation and the use of antibiotics will lead to the imbalance of intestinal microbiota and further cause gastrointestinal dysfunction. Regulating the structural disorder of intestinal microbiota can effectively inhibit the secondary injury after SCI, improve function, treat gastrointestinal dysfunction, and have a positive effect on urinary tract infection, which is undoubtedly very important for treating complications and the improvement of patients’ quality of life. Melatonin not only effectively blocks the secondary injury process after SCI, but also promotes the recovery of intestinal microbiota structure. It plays a synergistic role in intestinal microbiota to improve the prognosis of SCI.

Melatonin plays an important role in the treatment of acute and subacute SCI, including anti-apoptosis, anti-inflammation, anti-free radical damage, alleviating edema, protecting BSCB, promoting neurons, reducing the accumulation of astrocytes, and further affecting disease’s development in the chronic phase, such as regulating chronic inflammation and reducing scar formation. Intestinal microbiota can promote neuronal survival and axonal regeneration, secrete SCFA to regulate inflammation and immune function, and has positive significance for the treatment of complications after SCI.

There is no direct research proving that melatonin and intestinal microbiota have synergistic effects on the treatment of SCI. However, some studies have proved the synergy between the two from the side. Intestinal microbiota down regulates NF-κB pathway by short-chain fatty acids in SCI. Melatonin is also proved that down-regulate the signal expression of the same pathway. On the other hand, melatonin can modulate the intestinal barrier and motor function to regulate the structure of the intestinal flora. In addition, some studies have confirmed that the positive effect of melatonin on SCI is reduced after the use of antibiotics. It also indirectly shows that the two have a synergistic effect on the recovery of SCI. Therefore, melatonin and gut microbiota may act synergistically in the treatment of SCI and further studies will be needed to confirm their role and underlying mechanisms.

The disorder of intestinal microbiota after SCI occurs in the acute stage. However, in the current study, FMT mostly occurs in the chronic stage after SCI. Therefore, whether early use of FMT and melatonin to regulate the structure of intestinal microbiota and produce significant therapeutic effects on SCI is worthy of further study.

In addition, Majidpoor et al. found that different sensitivity of melatonin treatment in different injured segments (Majidpoor et al., 2021). Few study has reached similar conclusions, so the underlying mechanism deserves further study.

The intestinal tract contains a variety of flora and abundant lymphoid tissue, which has a strong immunomodulatory function. Gut-derived Treg cells have been confirmed to feature in the prognosis of stroke, and studies have confirmed that programmed death protein 1 (PD-1) and CC-chemokine ligand 28 (CCL28) regulate Treg cells (Wang P. et al., 2019; He et al., 2021). In addition, melatonin also has a regulatory effect on Treg cells (Álvarez-Sánchez et al., 2015; Glebezdina et al., 2019). Therefore, the combined effect of melatonin and gut microbiota whether further affect the prognosis of SCI through Treg cells is worthy of in-depth study.

This review discusses the relationship among melatonin, intestinal microbiota and SCI, and summarizes the progress of melatonin and intestinal microbiota in treating SCI. By elucidating the link among melatonin, the brain-gut axis and SCI, new insights and ideas for the treatment of SCI are provided, furthering the development of SCI treatment.

## Author contributions

YZ, YY, CB, and WD conceived the perspective of the work. YZ, RL, and SG drafted the manuscript. HL and CL designed the figures. All authors revised and approved the final version of the manuscript.
